# Editorial: Cybersecurity and artificial intelligence: advances, challenges, opportunities, threats

**DOI:** 10.3389/fdata.2024.1537878

**Published:** 2025-01-07

**Authors:** Pavlos Papadopoulos, Sokratis Katsikas, Nikolaos Pitropakis

**Affiliations:** ^1^School of Computing, Engineering and the Built Environment, Edinburgh Napier University, Edinburgh, United Kingdom; ^2^Department of Information Security and Communication Technology, Norwegian University of Science and Technology, Gjøvik, Norway

**Keywords:** cybersecurity (CS), artificial intelligence, adversarial machine learning, advances, challenges, opportunities, threats

## 1 Introduction

Cybersecurity and AI have become increasingly important in today's interconnected world. Developments in them and because of them find their way regularly not only to scientific literature but to the media as well. Despite its benefits, the increasing use of and reliance on AI, including Machine Learning (ML), have created a complex threat landscape. Simultaneously, cybercriminals have become more sophisticated and organized, targeting not only individuals but also organizations and even nations. These threat actors use advanced tactics, techniques, and procedures to exploit vulnerabilities to attack AI-powered systems. To mitigate these threats, it is essential to enhance our understanding of the risks involved and to develop effective, specially targeted cybersecurity strategies and robust defensive mechanisms. The increasing pervasiveness of AI and related technologies, coupled with the growing sophistication of cybercriminals, has created a challenging threat landscape that requires innovative solutions to mitigate.

## 2 Overview of published articles

Five articles are published in this Research Topic. They present novel research results in three areas, namely on ML-based clustering algorithms, on the trustworthiness of AI-based systems, and on risks emerging from the malicious use of AI.

Streaming services are highly popular today. The clustering of users of online or offline services is frequently used for fraud detection. Density-based spatial clustering of applications with noise (DBSCAN) is a clustering algorithm used in machine learning to partition data into clusters based on their distance to other points. Mochurad et al., in their article entitled “*A fast parallelized DBSCAN algorithm based on OpenMp for detection of criminals on streaming services*,” propose a parallel algorithm for fast clustering of users of a streaming service. The proposed algorithm maintains DBSCAN's distinctive features while exhibiting significant speed-up.

Within a Security Information and Event Management (SIEM) system, the User and Entity Behavior Analytics (UEBA) engine aims to analyze the behavior of employees, third-party contractors, and collaborators of the organization to detect misbehavior in user activities. In their article entitled “*A comprehensive investigation of clustering algorithms for User and Entity Behavior Analytics*,” Artioli et al. conduct a comprehensive investigation of multiple clustering algorithms, encompassing a wide range of clustering techniques, including traditional and more modern advances, and they evaluate their applicability for UEBA.

Current AI risk management frameworks neglect human factors, and metrics for socially related or human threats are missing. Polemi et al., in their article entitled “*Challenges and efforts in managing AI trustworthiness risks: a state of knowledge*,” provide a comprehensive approach to AI trustworthiness, combining technical and social mitigation measures, standards, and ongoing research initiatives by exploring various dimensions of trustworthiness of AI-based systems, covering legislation, AI cyber threat intelligence, and characteristics of AI adversaries. Furthermore, the socio-psychological threats associated with AI integration into society are examined, addressing issues such as bias, misinformation, and privacy erosion. As a result, the importance of collaboration between cybersecurity engineers, AI experts, and social-psychology-behavior-ethics professionals is highlighted.

AI is increasingly used to support the development of software. Even though this practice can improve coding proficiency, security of the developed code cannot be ensured. In fact, recent research has demonstrated that some AI models produce software with vulnerabilities. Negri-Ribalta et al., in their article entitled “*A systematic literature review on the impact of AI models on the security of code generation*”, conduct a systematic literature review to establish and systematize the state of the art on how AI models impact software security.

Recent advancements in AI, especially deep learning, have contributed to a significant increase in the creation of new realistic-looking synthetic media (video, image, and audio) and manipulation of existing media; this has resulted in what is currently called “deepfake” material. Altuncu et al., in their article entitled “*Deepfake: definitions, performance metrics and standards, datasets, and a meta-review*”, provide a comprehensive overview of deepfake, covering multiple important aspects of this emerging concept. Such aspects include different definitions of the concept, commonly used performance metrics and standards, and deepfake-related datasets. The paper also provides a meta-review of 15 deepfake-related survey papers published since 2020, focusing not only on the above aspects but also on the analysis of key challenges and recommendations.

We hope that the reader will find useful information in this Research Topic on some aspects of the emerging field of the intersection between AI and cybersecurity. Further research in this area is vital if we are to better understand the nature of AI as a “double-edged sword” and mitigate the accordant emerging risks.

